# Supplementary material for: Long-distance propagation of short-wavelength spin waves
Source: Nat Commun. 2018 Feb 21;9:738. doi: 10.1038/s41467-018-03199-8 (PMC5821877; doi:10.1038/s41467-018-03199-8)
Supplement: Supplementary file 1 — Supplementary Information [file 41467_2018_3199_MOESM1_ESM.pdf]

Long-distance propagation of short-wavelength spin waves

Liu et al.

Supplementary Note 1. Characterization of the YIG thin film

Supplementary fig. 1 shows the characterization of the 20-nm-thick YIG film used for device fabrication. Supplementary fig. 1d shows the FMR linewidth as a function of frequency, from which one can estimate the damping property of the film by using:

$$\Delta H_{\mathrm{FMR}} = \frac{2\alpha}{\sqrt{3}|\gamma|}\frac{\omega}{2\pi} + \Delta H_0 \qquad (1)$$

where ΔH_0 is the film inhomogeneity line broadening. The fitting curve gives $\alpha = 8.0 \pm 1.1 \times 10^{-5}$ and $\Delta H_0 = 4.2 \pm 0.07$ Oe.

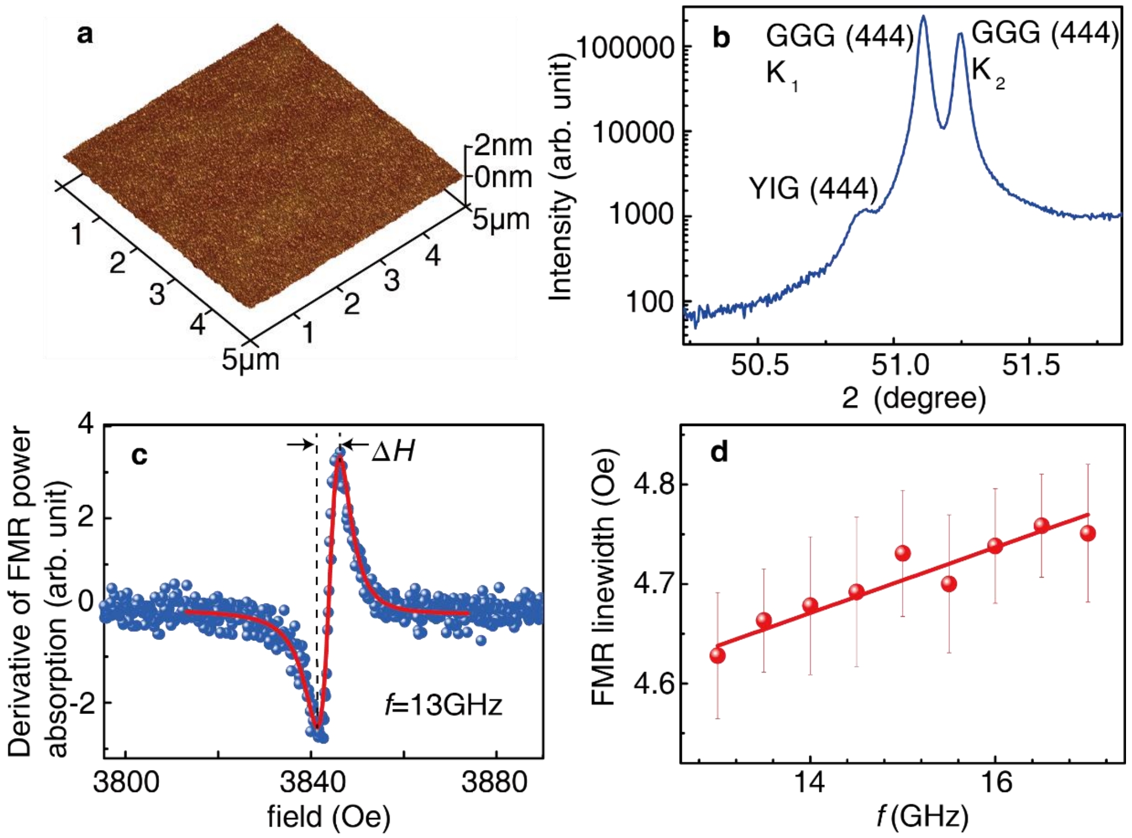

Supplementary Figure 1. Properties of 20-nm-thick YIG film fabricated by sputtering. a, measured atomic force microscope (AFM) surface image of the YIG thin film. Additional AFM data indicate that the film has an rms surface roughness of 0.067 ± 0.01 nm. **b,** measured XRD spectrum of the YIG thin film. The data confirm the existence of the YIG phase in the sample and indicate the (111) orientation of the film. **c,** FMR data obtained from the same YIG film. The circles give an FMR profile measured at 13 GHz, while the curve shows a fit to the derivative of a Lorentzian trial function. The fitting yielded $\Delta H = 4.7$ Oe. **d,** ΔH as a function of $\omega/2\pi$. The dots

are data extracted from experiments and the line shows a linear fit using Eq. 1. The error bars indicate the standard errors by the fitting of the derivative of a Lorentzian trial function in **c**.

Supplementary Note 2. Energy dispersive X-ray spectroscopy image

Supplementary fig. 2 shows the energy dispersive X-ray (EDX) spectroscopy image of Co/Ti/YIG structure. The cobalt, titanium and yttrium are selectively detected and highlighted by different colors. A Co/Ti/YIG spin-valve trilayer structure is clearly observed. Slight diffusion of Ti into Co and YIG might exist indicated by the color-coded image.

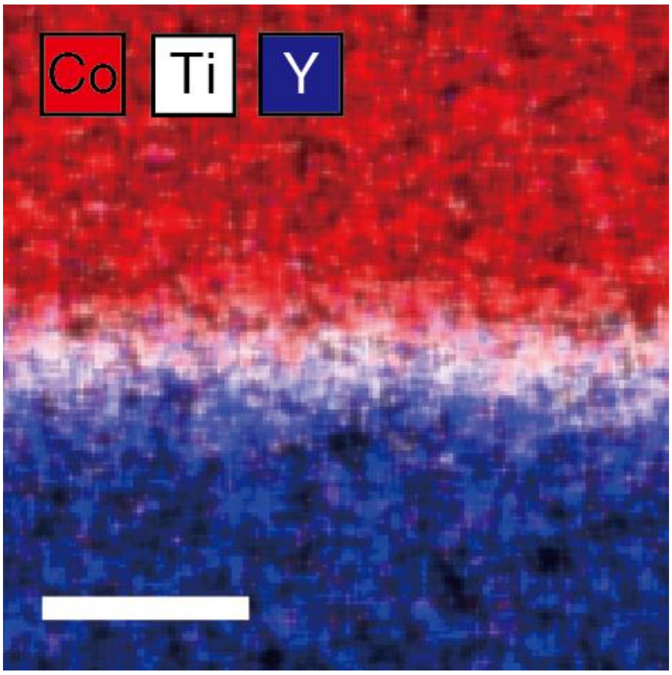

Supplementary Figure 2. Energy dispersive X-ray spectroscopy image corresponding to Figure 1e. Co/Ti/YIG spin-valve structure. Red: Cobalt; White: Titanium; Blue: Yttrium. The scale bar is 2 nm.

Supplementary Note 3. Minor loop measurement of Co-based device A2

After saturate all Co nanowires in one direction, the external field is ramped up to R state and then sweep back. The spectra taken at field smaller than 230 Oe shows two co-existing Co modes with positive and negative slopes. These two modes represent the resonances of Co nanowires saturated in

the +*y* or -*y* orientation respectively. All the nanowires are aligned into the same magnetization orientation when the field sweep down to -230 Oe.

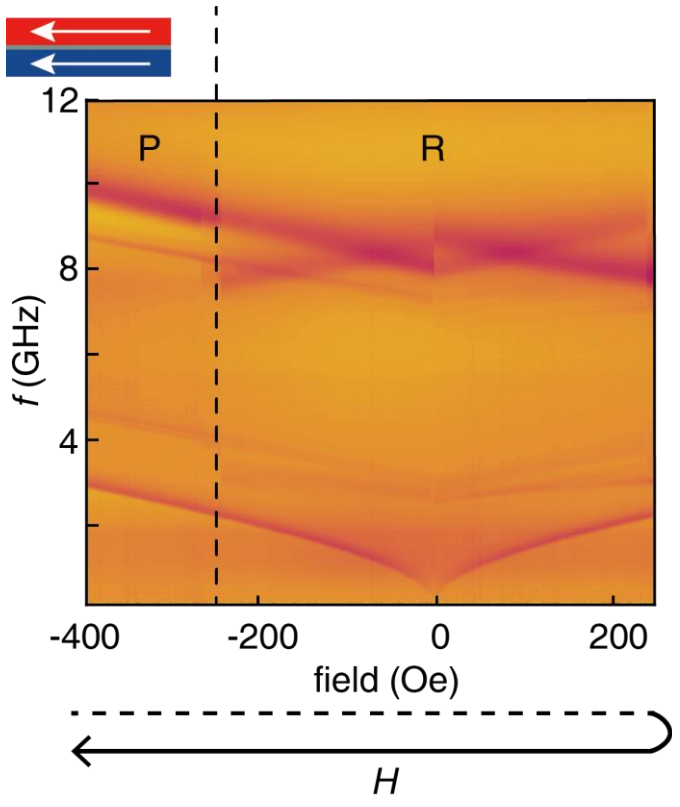

Supplementary Figure 3. Minor loop from the R state. Color-coded absorption spectra S_{11} in the reflection configuration measured with a minor loop field sweep: The field is first saturated at -3,000 Oe and swept from 230 Oe (R state) and back to -400 Oe with field steps of -2.5 Oe.

Supplementary Note 4. PSWSW spectra of the Co-based device A3

Supplementary fig. 4 shows the PSWSW spectra of the Co-based device (A3) with the longest propagation distance observed so far up to 60 μm. One can see a very clear propagation spin wave signal with phase information. The high *k* mode with *n*=4 is observed.

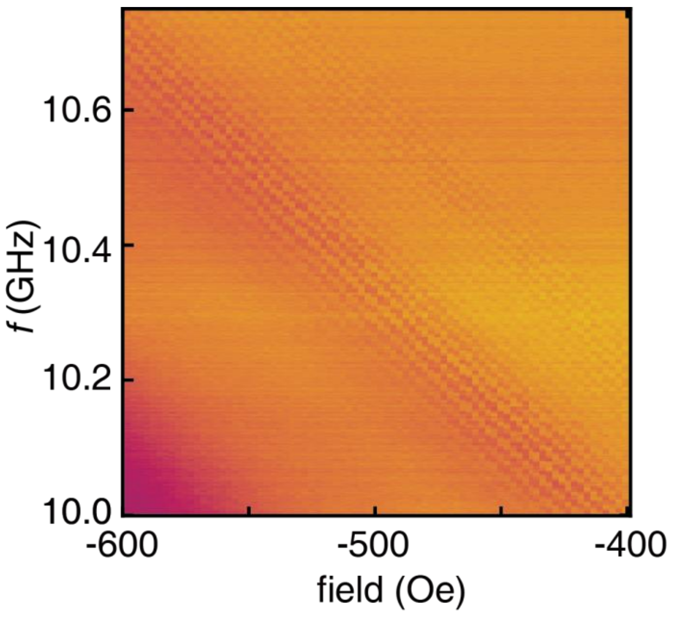

Supplementary Figure 4. Color-coded transmission spectra S_{12} measured on the 180 nm period magnonic spin-valve nanowires sample with $s = 60\ \mu\text{m}$ of device A3. The plot shows the *n*=4 mode. The field is first saturated at -3,000 Oe and swept from -600 Oe to -400 Oe with field steps of 2.5 Oe.

Supplementary Note 5. Spin wave amplitude of different modes

Supplementary fig. 5 shows the spin wave amplitude of different modes as a function of external field on Co-based device (A2). One can observe that *n*=4 mode exhibit a very strong spin wave amplitude in the high external field even compared to k_1 mode which is excited by CPW. The spin wave amplitude of *n*=4 mode increase with the applied external field.

The decay length of the propagating spin wave is given by the equation:

$$l_d = \frac{v_g}{2\pi\alpha f_0} \qquad (2)$$

Where v_g is the spin wave group velocity, α is the damping parameter and f_0 is the spin wave resonance frequency.

We take sample A2 for an example and the measured PSWSW efficiency is 106% shown in Table 1. Taking into account of $s = 30\ \mu\text{m}$ and a decay length of $300\ \mu\text{m}$ calculated by Eq. 2 for n=4 mode, the spin wave amplitude excited at $s = 0\ \mu\text{m}$ is 1.105 times larger at the excitation than the detection. We also calculate the same ratio of CPW excited mode at 100 Oe which is 1.014. The calculated PSWSW efficiency subtracting the influence of the spin-wave group velocity is therefore 116%, even larger than the PSWSW efficiency listed in Table 1.

It is worth noticing that here the PSWSW efficiencies shown in main text and Table 1 are relative values. It is challenging to precisely determine an absolute excitation efficiency because of the difficulty in estimating the microwave power dissipation in the CPW and the non-reciprocal propagation. The power level at the S_{11} baseline suggests a reflected power when the hybrid structure is off the resonance (R_{off}). As soon as the resonance condition is met, more power is absorbed by the spin-wave excitation of the hybrid structures and therefore less reflection power is detected by the VNA (R_{on}). To have a rough estimation, we consider the absolute excitation efficiency to be $\eta_{\text{abs.}} = (R_{\text{off}} - R_{\text{on}}) / R_{\text{off}}$. By this way, the absolute excitation efficiency of the CPW mode, n=2 PSWSW mode, n=4 PSWSW mode at -400Oe can be estimated as 5.69%, 1.57%, 5.4%. Nevertheless, such consideration ignores other power loss, such as heat dissipation and impedance mismatching, and therefore is only a rough estimation.

There are a few of PSWSW modes which have not been observed in the experiment such as n=2 mode in sample A3 and B1 shown in Table 1. This does not mean these modes do not exist but rather the signal is so small to detect. This can be due to many reasons. Firstly, the excitation efficiency can be varied between different modes, e.g. if the mode is very far from the resonances of the wires, then the excitation efficiency might be considerably lower than that of the modes close to

wire resonances. Take sample B1 for instance, the modes n=6 and n=8 are close Ni wire resonances and therefore show sizable signals, whereas n=4 and n=10 modes are weaker but still detectable with reasonable signal strength. However, if the frequency is too far away such as n=2 and n=12, the signal can hardly be detected. Therefore, only n=4,6,8,10 are written in Table 1. Also, the decay length is strongly dependent on the resonance frequencies as shown in the Eq. 2 which indicates that the higher the frequency is, the shorter the decay length becomes and consequently the transmission signal is attenuated more. This also explains why in general high order PSWSWs are very hard to detect. What we are looking at is the transmission signal of PSWSWs, and therefore the detection distance plays a critical role in the experiment. For example, sample A3 has the longest detection distance, i.e. the distance between two integrated CPW antennas. The transmission is expected to be weaker and as a result, only the mode with the highest excitation efficiency and good decay length can be detected, which is n=4 mode in this case. Nevertheless, we do see other modes such as n=2 and n=6 in the reflection spectra S_{11}.

For n=2 and n=4 modes, the group velocities v_g extracted from experimental data agree well with the theoretical values. However, there is a small departure for the n=6 mode. This small disagreement may be an influence of the standing waves across the YIG film thickness, which is negligible for spin waves with relatively long wavelengths but not for those with wavelengths comparable with the film thickness. Future work is of interest that examines this possibility through measurements using YIG films of different thicknesses.

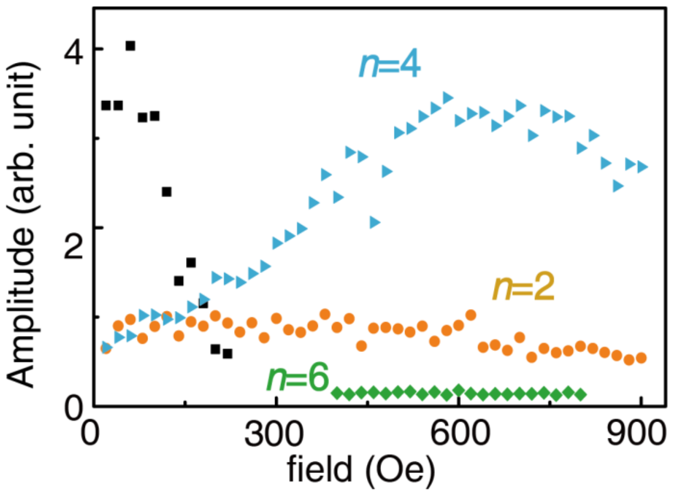

Supplementary Figure 5. Propagating spin wave amplitude of S_{12} as a function of the external magnetic field. Spin wave amplitude of different spin wave modes of the Co-based device (A2). Black squares: CPW-excited spin wave amplitude in the 20 nm-thick plain YIG film extracted from experimental data. PSWSW amplitude extracted from experimental data with mode number *n*=2 (yellow circles), *n*=4 (blue triangles) and *n*=6 (green diamonds).

Supplementary Note 6. PSWSW spectra of the CoFe-based device C1

Supplementary fig. 6 shows the PSWSW spectra of the CoFe-based device C1 with mode number *n*=8 in the low field region. A strong spin wave signal starting from zero field to 200 Oe is observed in the high frequency up to 23 GHz whose wavelength is down to 50 nm.

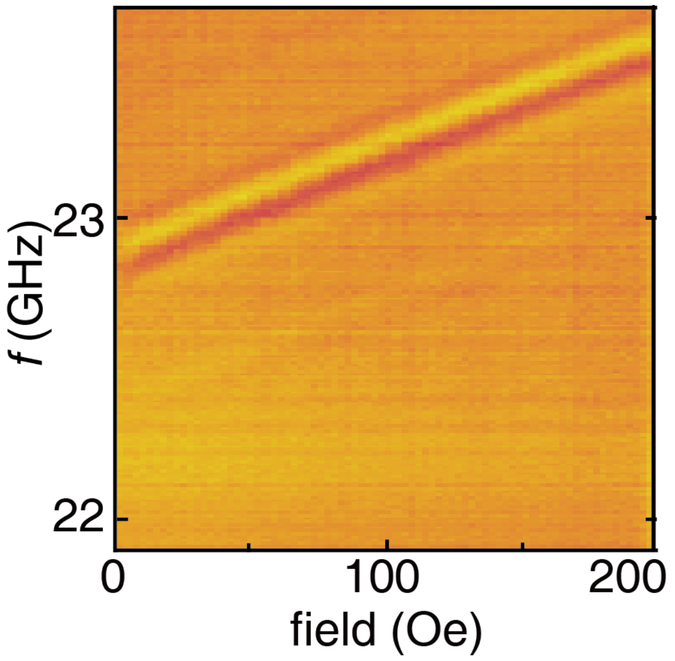

Supplementary Figure 6. Color-coded plot of transmission spectra S_{21} measured on the CoFe-based 200 nm period magnonic spin-valve nanowires sample device C1 with $s = 15\ \mu m$ in the P state. The field is first saturated at 3,000 Oe and then swept from 200 Oe to 0 Oe with field steps of -2.5 Oe.

Supplementary Note 7. Vibrating sample magnetometer measurements of reference samples

Supplementary fig. 7 shows the vibrating sample magnetometer (VSM) measurements of different reference samples. The 20 nm thin film YIG, 25 nm thin film Co and trilayer thin film with Co (25)/Ti (1)/YIG (20) (in nm) have been studied. One can observe that the black loop is not simply the superposition of the red and blue loops. A coercive field generated by the dipolar coupling between Co and YIG causes a delay of the reversal of Co magnetization in the hysteresis loop.

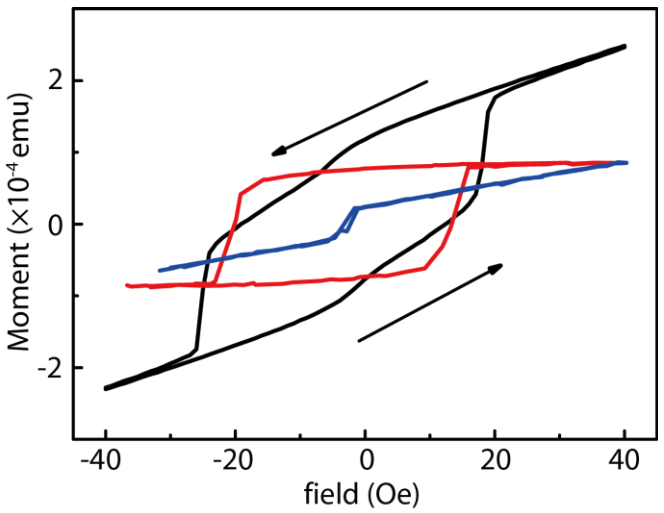

Supplementary Figure 7. VSM magnetization loops of three samples with different kinds of magnetic layers. The three samples are 20 nm thin film YIG (blue line), 25 nm thin film Co (red line) and trilayer thin film with Co (25)/Ti (1)/YIG (20) (in nm) (black line), respectively. The magnetic field is applied in the film plane.

Supplementary Note 8. Simulated dispersion relation for SWs with a ferromagnetic exchange coupling

Supplementary fig. 8 displays the simulated dispersion relation for SWs with a ferromagnetic ($A = 4.875 \times 10^{-13}$ J m^{-1}) exchange coupling between the stripe and the YIG at a bias field of 50 Oe. In (a) the magnetization in the stripes and the YIG are both aligned in the direction of the external bias field forming the P configuration. On the contrary in (b) the magnetization of the stripes is reversed, opposing the external field direction and leading to the AP configuration (YIG magnetization is still pointing in field direction). In both cases the position of the excited SW-modes is shifted due to the additional exchange interaction. While the P state leads to a shift to higher frequencies, the AP configuration decreases the frequencies of the excited modes. This behavior is in good agreement with the observed experimental results which display a frequency shift at the switching of the YIG

magnetization. The reason is again the dispersion relation underneath the strips, which is different depending on whether they are in the P or AP configuration.

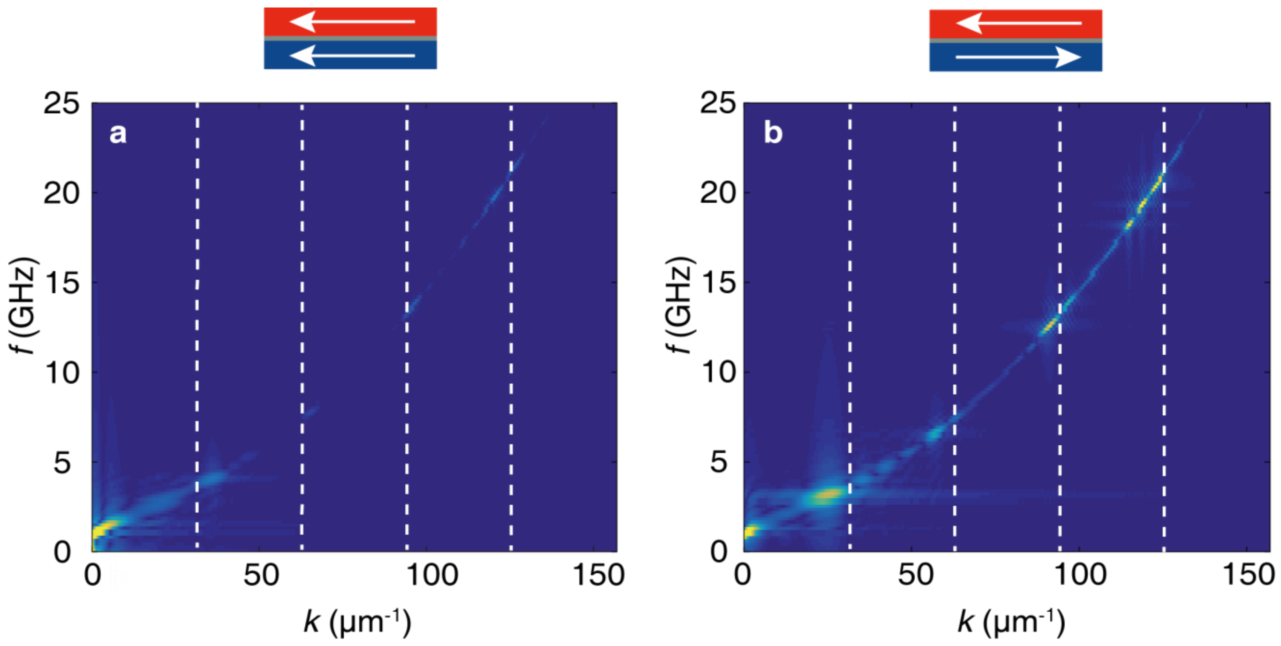

Supplementary Figure 8. Dispersion relation from simulation considering interlayer exchange coupling. Simulation structure is with a period of 200 nm (stripe width 100 nm, gap 100 nm), an external bias field of 50 Oe and an exchange coupling ($A = 4.875 \times 10^{-13}\ \mathrm{J\ m^{-1}}$) between the YIG and the stripes, in the P (**a**) and AP (**b**) configurations.

Supplementary Note 9. PSWSW spectra of the CoFe-based devices C1 and C2

Supplementary fig. 9 shows the PSWSW spectra of the CoFe-based devices C1 and C2 with mode number n=4. Supplementary fig. 9 a and b show the result on device C1 where interlayer exchange coupling is expected. As a result, a blueshift (a) or redshift (b) of spin wave resonance frequency is observed in P and AP states respectively. This observation can be understood as the result of the modified exchange constant λ_{ex} induced by the interlayer exchange coupling in the magnonic spin-valve hybrid structure. A thick Al_2O_3 layer between Co and YIG layers was used in device C2 to

eliminate the interlayer exchange coupling. With only dipolar-dipolar interaction, one also observes the high order mode spin wave but no spin wave frequency shift is found in Supplementary fig. 9 c and d. This result agrees well with the magnetic simulation shown in Supplementary fig. 8.

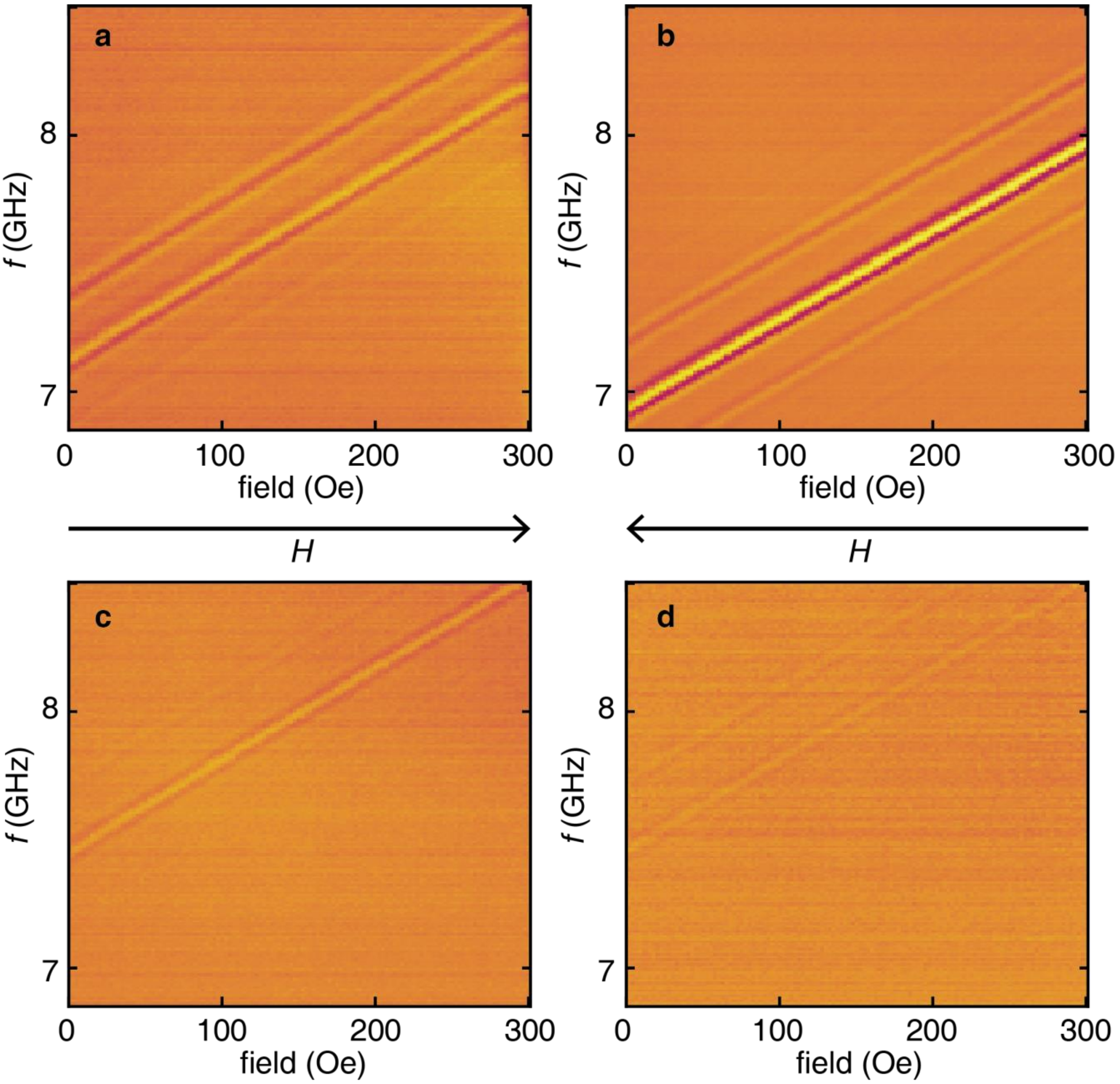

Supplementary Figure 9. Color-coded plot of transmission spectra S_{21} measured on the CoFe-based 200 nm period magnonic spin-valve nanowires sample C1 and C2 with $s = 15$ μm. These four figures show the *n*=4 spin wave mode in the same frequency range. a and **b** are measurements of sample C1. **c** and **d** are measurements of C2 whose interlayer is replaced by a thick Al_2O_3 layer and therefore the interlayer exchange coupling is removed. **a** and **c**, the field is first saturated at 3,000 Oe and then swept from 300 Oe to

0 Oe with field steps of -2.5 Oe. **b** and **d,** the field is first saturated at -3,000 Oe and then swept from 0 Oe to 300 Oe with field steps of 2.5 Oe.

Supplementary Note 10. PSWSW spectra of the Co-base devices A4)

Supplementary fig. 10 shows the PSWSW spectra of the Co-base 600 nm period magnonic spin-valve nanowires devices A4 with mode number n=14. A 7 nm-thick Al_2O_3 layer between Co and YIG layers was inserted in device A4 to eliminate the interlayer exchange coupling. This insulating layer was grown by electron beam evaporation before Co is deposited without breaking the vacuum in the chamber. Only n=14 PSWSW mode is observed in the transmission spectra. And the intensity of this mode is much weaker compared to the PSWSW mode observed in the other Co, Ni, and CoFe-base device with Ti spacer. Other PSWSW modes are observed in the reflection spectra with low intensities. With merely dipolar-dipolar interaction the device A4 shows a much lower excitation efficiency of PSWSW.

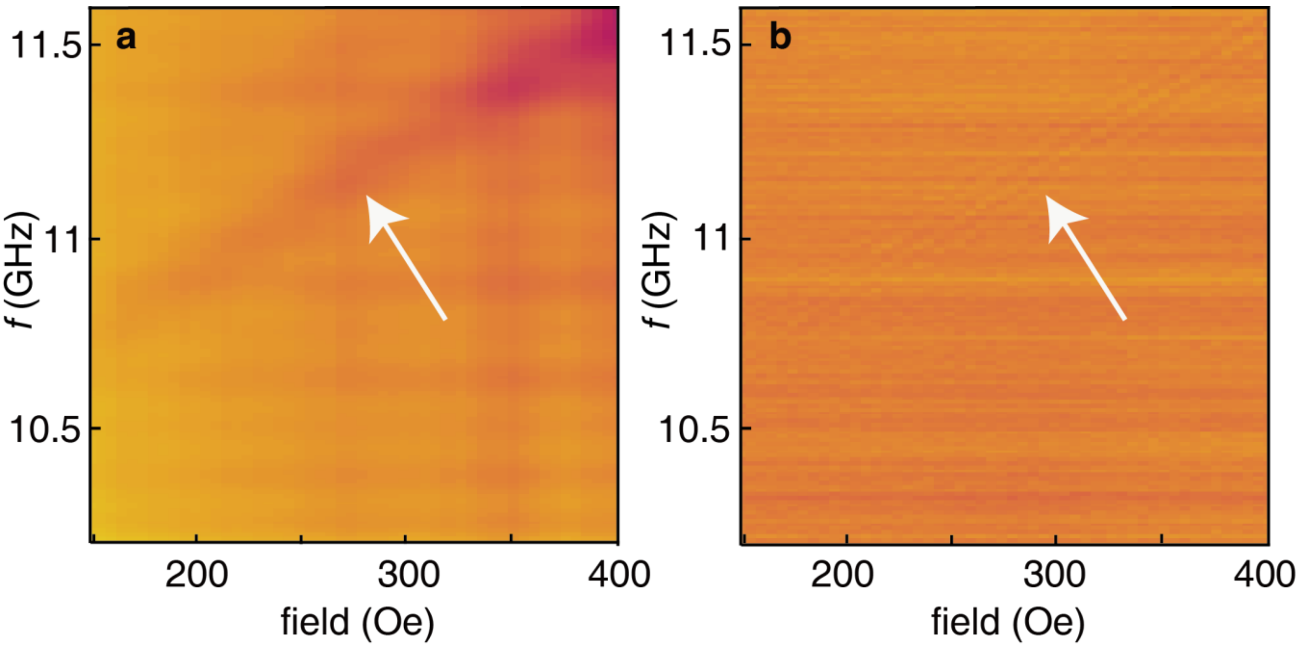

Supplementary Figure 10. Color-coded plot of reflection spectra S_{11} and transmission spectra S_{12}

measured on the Co-based 600 nm period magnonic spin-valve nanowire device A4 with 7 nm Al_2O_3 middle layer between YIG and Co. The propagation distance $s = 30\ \mu\text{m}$. These two figures show the *n*=14 spin wave mode in the same frequency range. In this sample the spacer is replaced by a 7nm Al_2O_3 layer and therefore the interlayer exchange coupling is eliminated. The field is first saturated at -3,000 Oe and then swept from 150 Oe to 400 Oe with field steps of 5 Oe.

Supplementary Note 11. Simulated dispersion relation while shrinking Co nanowires

Supplementary fig. 11 shows the result of a simulation with a Co stripe period of 20 nm (stripe width 5 nm, gap 15 nm). There are now 18 stripes with dimensions of 1 μm×5 nm×5 nm. Additionally, dimensions of the YIG waveguide were reduced to 12 μm×1 μm×5 nm. One can observe a clear excitation of SWs at the k-vectors associated with the period of the stripes. The two dashed lines indicate the *n*=2 and *n*=4 PSWSWs respectively. There is a low-frequency excitation at the resonance of the magnetic stripes in the YIG dispersion curve because when the stripes are at the resonance, a strong dynamic coupling affects the precession of YIG.

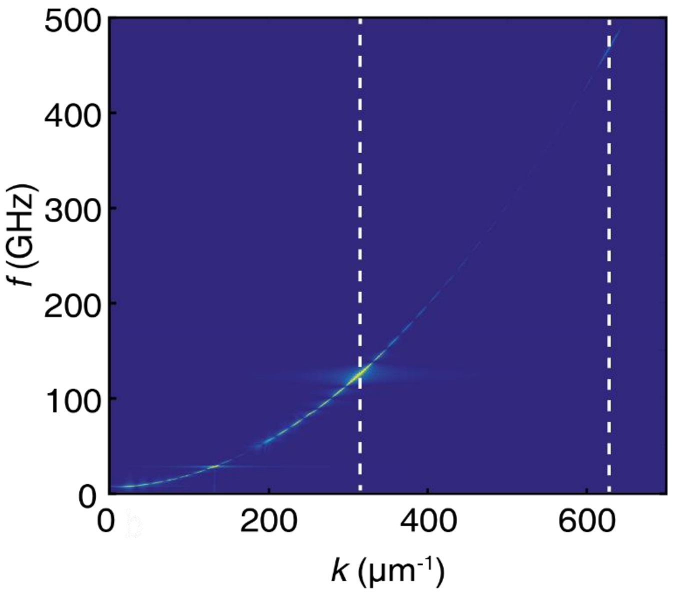

Supplementary Figure 11. Dispersion relation up to sub-THz. Micromagnetic simulations on device with nanowire array period of 20 nm (stripe width 5 nm, gap 15 nm) under an external bias field of 100 mT.
